# Spatiotemporal variation characteristics of livestock manure nutrient in the soil environment of the Yangtze River Delta from 1980 to 2018

**DOI:** 10.1038/s41598-022-11267-9

**Published:** 2022-05-05

**Authors:** Shuyan Zhao, Yujiao Chen, Xiaomin Gu, Mengru Zheng, Ziyi Fan, Dan Luo, Kaihong Luo, Bo Liu

**Affiliations:** School of Geographic Science, Nantong University, Nantong, 26000 China

**Keywords:** Ecology, Environmental sciences

## Abstract

The pollution problem caused by surplus livestock manure nutrient is becoming more and more serious; thus, analyzing characteristics of the temporal and spatial patterns of livestock manure nutrient and assessing the risks are extremely important. The pollution coefficient method was used to figure out the spatiotemporal variation characteristics of the nitrogen (N) and phosphorus (P) emissions of livestock manure nutrient in soil of the Yangtze River Delta from 1980 to 2018. High-risk areas for livestock manure pollution were determined by matching degree assessment between livestock manure nutrient supply and crop nutrient requirements. Clustering and principal component analysis were applied to select main control factors. The results found that the total discharge and manure N and P loads in animal manure nutrient showed an overall trend of increasing first and then decreasing in the Yangtze River Delta in the soil from 1980 to 2018. The center of manure N and P loads shifted from the central region to the northeastern and southwestern regions. There were four typical patterns for manure N emissions, the main control factors were meat production and primary industry. Meanwhile, the main control factors for the four typical patterns of manure P emissions were meat production and cultivated land area. This research provides a theoretical basis for the sustainable development of the livestock breeding industry and is of significance for promoting a balance between planting and breeding in the Yangtze River Delta.

## Introduction

China produces substantial amounts of nutrient from livestock grazing as one of the world’s largest livestock producers^1^. The total N (TN) and the total P (TP) emissions from the livestock breeding industry reached 596,000 tons and 120 thousand tons, respectively, in 2017 according to the “Second National Pollution Source Census Bulletin”^2^. In China, however, the usage efficiency of livestock manure is less than 60%^3^. Surplus livestock manure nutrient are discharged into water bodies, causing serious environmental problems such as eutrophication and destruction of the underwater ecosystem^4^. Furthermore, excessive chemical fertilizer application accompanied with a high rate of nutrient loss of 20–36% further exacerbate nonpoint-source pollution risks in China as compared with the fertilizer input in most developed regions, such as Euro-American major countries^5–7^. The rapid development of the livestock breeding industry and excessive application of chemical fertilizers in the Yangtze River Delta have resulted in excess nutrients, leading to side effects such as inhibition of crop growth^8^, increasing risks of pollution to water and soil^9^. Thus, the use of manure to replace chemical fertilizer is an effective approach for recycling nutrient and slowing soil deterioration^10^. Understanding the spatiotemporal evolution of manure and its key drivers are crucial for promoting the balance of planting and breeding, solving the problems of nutrient surplus from manure, reducing chemical fertilizer usage, and promoting sustainable development of the livestock industry in the Yangtze River Delta^11^. However, the spatiotemporal evolution of manure and its key drivers have not been well documented in this region.

Manure nutrient have been considered necessary in investigations of water quality and watershed biogeochemistry^12^. A variety of methods have been adopted to study the nutrient and load of livestock manure. For example, Yan et al.^13^ used spatial autocorrelation analysis method developed the spatial pattern and manure N load evolution of farmland livestock farming waste in Anhui Province, China; Li et al.^9^ used nutrient balance and risk of N and P pollution in livestock manure methods evaluated environmental impact based on temporal and spatial analysis. Globally, Mekonnen et al.^14^ estimated the anthropogenic P load and related grey water footprints (GWFs) from 2002 to 2010 and assessed the water pollution level (WPL) related to P. However, these studies mostly carried out at a national level within a relatively short time period (less than 20 years). Temporal and spatial variation of livestock manure nutrient at a region and on a long-term scale are needed to better understanding manure nutrient distribution characteristics in-depth, which is essential for the sustainable development of livestock breeding^15^.

The main aims of this research were as follows: (1) to clarify the temporal and spatial variation characteristics of livestock manure N and P load in the Yangtze River Delta and determine high-risk areas for livestock manure pollution, (2) to select and establish typical models of N and P emissions, and (3) to identify the main factors affecting the typical models based on principal component analysis. This research is of great importance for promoting the balance of planting and breeding, and solving the nutrient surplus from livestock manure in the Yangtze River Delta.

## Materials and methods

### Overview of the study area

The Yangtze River Delta region was used as the research area in this study. This area comprises 16 cities: eight in Jiangsu (Nanjing, Yangzhou, Taizhou, Nantong, Zhenjiang, Changzhou, Wuxi, and Suzhou), seven in Zhejiang (Hangzhou, Huzhou, Jiaxing, Shaoxing, Ningbo, Zhoushan and Tai), and Shanghai^16–18^. The study period ranged from 1980 to 2018. During this period, Yangzhou adjusted its administrative divisions. Taizhou was separated from Yangzhou and became a prefecture-level city (State Council^19^). Therefore, data for Taizhou prior to 1996 was included in Yangzhou.

### Calculation method

#### Emissions of livestock manure and urine, and manure N and P

Livestock manure emissions are typically estimated using the pollution coefficient method^20,21^. The pollution coefficient represents the annual excretion of livestock manure and pollutants and is calculated using the following formula:

1. Livestock manure (fresh quality):
  1 $$Q{M}_{i}={S}_{i}\times {P}_{i}\times {M}_{i}/1000,$$
  2 $$Q{U}_{i}={S}_{i}\times {P}_{i}\times {U}_{i}/1000,$$
  where *QM* and *QU* are excrement emissions and urine emissions, respectively, 10^7^ kg per year; *i* is the type of sow, hog, cattle, laying poultry, or meat poultry; *S* is the quantity of livestock produced during the year or inventory at the end of the year, 10^4^ head; *P* is the feeding cycle, d; *M* and *U* are the daily pollution coefficient of livestock excrement and urine, respectively, kg (d head)^−1^; 1000 is the conversion factor.

According to the main breeding purposes of livestock, meat livestock (hogs, meat poultry) was based on slaughter volume in the calculation, and livestock (sows, cattle, laying poultry) quantity for labor, eggs and milk, and reproduction were calculated based on stock volume^22^. According to the main breeding purpose of livestock and poultry, meat livestock and poultry (pork, meat and poultry) select their slaughter volume to participate in the calculation, and livestock and poultry (sows, cows, eggs and poultry) used for labor, egg and milk, and breeding purposes use their stock volume.

The feeding cycle of hogs, meat poultry, sows, cattle, and laying poultry were 180, 48, 365, 365, 365 days, respectively, following the data published by the State Environmental Protection Administration^23^. The livestock breeding quantities was obtained from the Statistical yearbooks for the Yangtze River Delta cities from 1980 to 2018.

The daily pollution coefficient for livestock excrement and urine refers to the quality of a single livestock’s daily excrement and urine. It was calculated using a weighted average method based on data collected from the following materials. Results are shown in Table 1.

(2) Livestock excrement and urine nutrients:
  3 $$T{N}_{i}={QM}_{i}\times {MN}_{i}+{QU}_{i}\times {UN}_{i},$$
  4 $$T{P}_{i}={QM}_{i}\times {MP}_{i}+{QU}_{i}\times {UP}_{i},$$
  where *TN* and *TP* are, respectively, the manure N and P emissions of livestock, 10^7^ kg per year; *QM* and *QU* are the amount of livestock excrement and urine, respectively, 10^7^ kg; *MN* and *MP* are, respectively, the nutrient content of excrement N and P in livestock, percentage (%); *UN* and *UP* are, respectively, the nutrient content of urine N and P in livestock, percentage (%).

Livestock manure N and P emissions estimation are based on fresh quality. Manure N and P emissions contents of various livestock were calculated using the weighted average method (Table 1).

**Table 1:** Daily pollution coefficient of livestock excrement and urine for each livestock (fresh quality).

Livestock	Manure/urine	Ranges	Weighted mean (kg (d head)^−1^)	Nutrient	Ranges	Weighted mean (%)
Sow	Excrement	1.58–3.29^24^, The First National Pollution Source Census Data Compilation^25^	2.44	N	0.24–2.96^26,27^	0.55
				P	0.09–1.76^27,28^	0.26
	Urine	5.06–5.48^24^, The First National Pollution Source Census Data Compilation^25^	5.27	N	0.17–0.50^26,27^	0.18
				P	0.02–0.15^26,27^	0.02
Hog	Excrement	0.64–12.00^26,27,29,30^	2.71	N	0.24–2.96^26,27,31^	0.55
				P	0.09–1.76^27,28^	0.26
	Urine	1.42–6.90^26,27,29^	3.86	N	0.17–0.50^26,27^	0.18
				P	0.02–0.15^26,27^	0.02
Cattle	Excrement	10.00–60.00^26,29^	24.87	N	0.30–0.84^26,28,29^	0.38
				P	0.02–0.41^28,29,31^	0.1
	Urine	3.00–26.71^26,27,29^	11.70	N	0.50–1.20^26,28,29^	0.51
				P	0.01–0.10^27–29^	0.02
Laying poultry	Excrement	0.01–0.66^26,27,32–35^	0.12	N	0.42–3.00^26,27,31^	0.81
				P	0.22–1.54^27,31,36^	0.37
Meat poultry	Excrement	0.06–0.22^37–40^	0.12	N	0.6–1.183^37–40^	0.76
				P	0.05–1.10^37–40^	0.34

#### Manure N and P loads

The N pollution load of livestock manure per unit of cultivated land area was calculated by divided the amount of livestock manure N emissions by the actual local cultivated land area. This quantitative indicator can indirectly estimate the pollution caused by local livestock and poultry breeding^41^. The manure N load of each city was calculated by dividing the total discharge of livestock manure nutrient by the corresponding sown area of crops for each city in the Yangtze River Delta. The calculation formula is as follows:

5 $${L}_{TN(TP)}=\frac{\sum {TN}_{i }({TP}_{i })}{A}\times {10}^{7},$$

where ${L}_{TN(TP)}$ is the manure N (P) load, kg hm^−2^; TN (TP) is the total amount of manure N (P), 10^7^ kg per year; A is the corresponding sown area of municipal crops, hm^2^; 10^7^ is the conversion factor. The sown area of crops in each city was taken from the statistical yearbooks of the Yangtze River Delta cities from 1980 to 2018.

#### Nutrient absorption capacity of land

The nutrient absorption capacity of land refers to the amount of nutrient applied to farmland without leading to nutrient accumulation. According to the “Technical Guidelines for Estimating Livestock Manure Land Carrying Capacity” (hereinafter referred to as the Guide), the formula for calculating the total N (P) nutrient requirements of the Yangtze River Delta plants is as follows:

6 $${A}_{n,i}=\sum ({P}_{r,i}\times {Q}_{i}\times {10}^{-2}),$$

where *A*_*n, i*_ represents the total plant N (P) nutrient requirements in the area, 10^3^ kg a^−1^; *P*_*r,i*_ represents the total output of the i crop in the region, 10^3^ kg a^−1^; *Q*_*i*_ represents the amount of N (P) required for 100 kg output of the i crop in the area, kg.

Crop types and output were obtained from the 2018 statistical yearbooks of cities in the Yangtze River Delta. Requirements for N and P nutrient per 100 kg of crop yield refer to the “Guide” (Table 2).

**Table 2:** Yield of main crops in the Yangtze River Delta in 2018, and the amount of N and P absorbed by 100 kg of yield.

Crop species	Total output/10^7^ kg	N removed for 100 kg production/kg × 100 kg^−1^	The amount of P removed for 100 kg production/kg × 100 kg^−1^
Rice	1287.74	2.20	0.80
Wheat	490.19	3.00	1.00
Beans	62.72	7.20	0.75
Potato	35.50	0.50	0.09
Corn	73.07	2.30	0.30
Oil	79.14	7.19	0.89
Vegetables	3232.95	0.28	0.09
Orchard	637.01	0.21	0.03
Cotton	1.88	11.70	3.04
Sugar	38.65	0.18	0.02

The crop nutrient demand from manure was calculated according to formula (7),

7 $${A}_{n,m}=\frac{{A}_{n,i}\times FP \times MP}{MR},$$

where *A*_*n,m*_ represents the nutrient demand of plants from livestock manure, 10^3^ kg a^−1^; *A*_*n,i*_ represents the total plant N (P) nutrient requirements in the area, 10^3^ kg a^−1^; *FP* represents the proportion of the total nutrient demand of crops supplied by fertilization, %; *MP* represents the ratio of livestock manure nutrient demand to the total fertilized nutrient provided under farmland fertilization management, percentage %; *MR* represents the utilization rate of manure in the current season, %.

According to the “Guide”, the utilization rate of manure N in the current season is 25%-30%, and the utilization rate of manure P in the current season is 30–35%, the utilization rate of manure N and P in the current season is 25% and 30%, respectively. According to Yu et al.^42^ and expert advice, the TN content of soil is 1.54 g kg^−1^ and the available P content is 44.72 mg kg^−1^, the soil N and P nutrient is at level I and the proportion supplied by fertilization is 35% (Table 3). The manure requirement of livestock accounts for 50% of the total fertilization^43^.

**Table 3:** Recommended ratio of fertilization and supply of nutrient under different soil nitrogen and phosphorus levels.

Soil nitrogen and phosphorus nutrient level		I	II	III
The proportion of the total nutrient demand of crops supplied by fertilization		35%	45%	55%
Soil total nitrogen content (g kg^−1^)	Dry land (field crops)	> 1.0	0.8–1.0	< 0.8
	Paddy field	> 1.2	1.0–1.2	< 1.0
	Vegetable field	> 1.2	1.0–1.2	< 1.0
	Orchard	> 1.0	0.8–1.0	< 0.8
Soil available phosphorus content (mg kg^−1^)		> 40	20–40	< 20

### Statistical analysis

All maps were created using Arcmap (v10.5, Esri Inc., www.esrichina.com.cn). In order to show spatial changes in the manure loads of livestock over the past 40 years in the soils more intuitively, we selected the years with the most apparent differences (1980, 1995, 2005, 2018) for nuclear density analysis in Arcmap 10.5 software.

Kernel density analysis is used to calculate the density of point elements around each output raster pixel. It can intuitively reflect the distribution of discrete measurement values in a continuous area, and the result can effectively display changes in the load center of gravity.

In this paper, the systematic cluster analysis and principal component analysis (PCA) in SPSS are used to analyze the factors affecting nitrogen and phosphorus emissions of livestock and poultry.

The Ward minimum variance method was applied to conduct variable analysis based on the similarity of manure N and P emissions in these cities^44^. The distance coefficient between groups was calculated using the binary European distance square method^45^. Correlation relationships between different variables (manure N and P emissions in the soil, the total output value of animal husbandry, population, gross domestic production (GDP), etc.) were evaluated by PCA in SPSS software (IBM Corp. Released 2013. IBM SPSS Statistics for Windows, Version 22.0. Armonk, NY: IBM Corp.)^46^.

## Results and discussion

### Spatiotemporal variations in N and P emissions from livestock manure

#### Manure N and P production and comparison with fertilizer usage in the Yangtze River Delta

**Figure 1:**
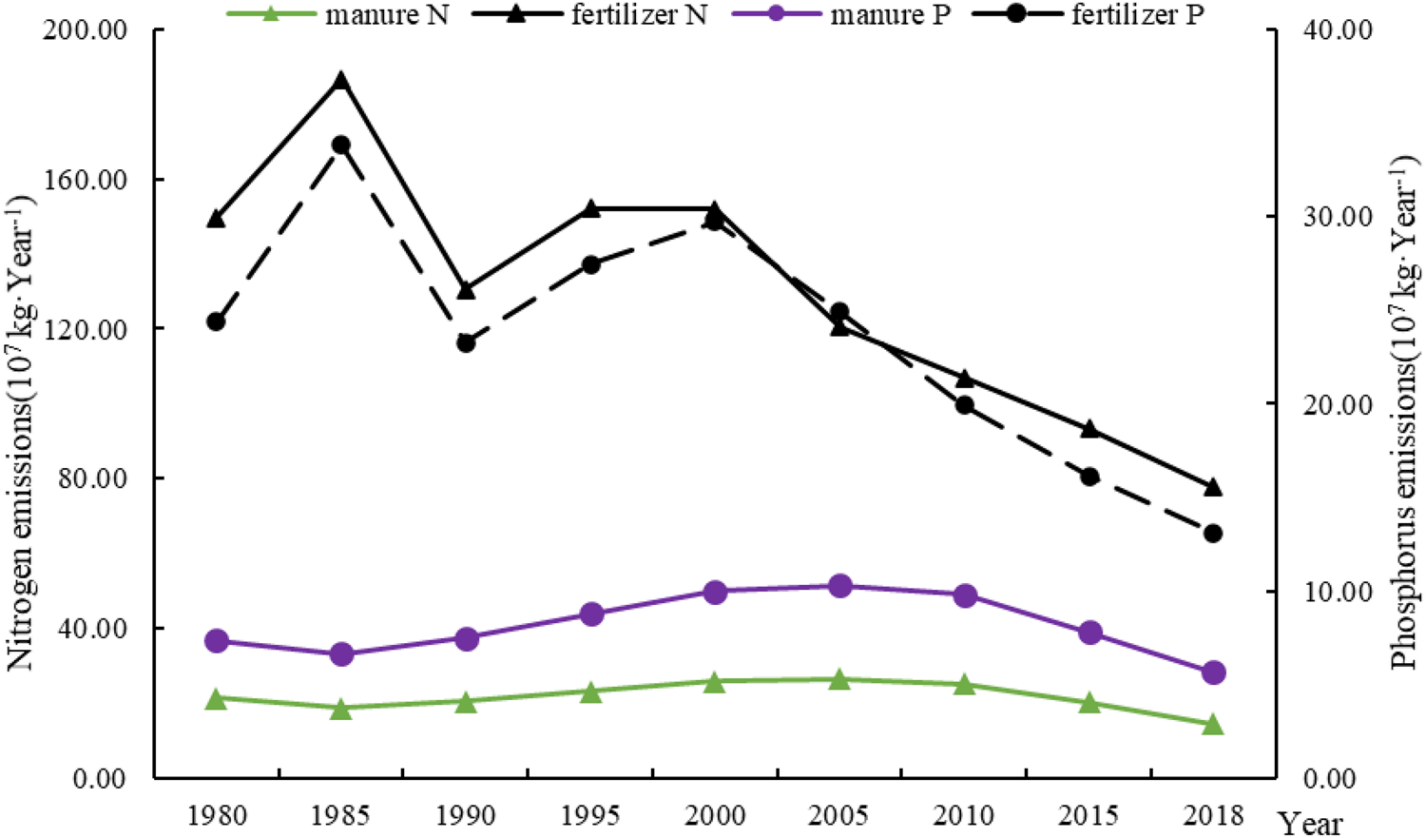
Application of N and P fertilizers and the amount of N and P emissions from organic fertilizers (manure) in the Yangtze River Delta from 1980 to 2018.

Although the amounts of chemical fertilizers used in the Yangtze River Delta region fluctuated greatly before 2000, the overall trend was still increasing, and there was a continuous downward trend since 2000 (Fig. 1). Specifically, the amount of chemical fertilizer used in the Yangtze River Delta region reached a peak of nearly 40 years in 1985, with 1.87 × 10^9^ kg of nitrogen fertilizer and 3.38 × 10^8^ kg of phosphate fertilizer. After entering the low period in 1990, it began to recover slowly, but since the beginning of the twenty-first century, the amount of fertilizer application has shown a continuous downward trend. By 2018, the amount of fertilizer nitrogen and phosphorus applied in the Yangtze River Delta region was 7.79 × 10^8^ kg and 1.31 × 10^8^ kg, respectively, which was a decrease of 58.28% and 61.28% compared to 1985.

The change trend of fertilizer application in the Yangtze River Delta may be a combined effect of the national policy and the change of cultivated land area. Since the 1970s, my country has vigorously promoted the fertilizer industry and agricultural fertilization^47,48^, which reached a peak in 1985. However, due to the adjustment of agricultural policies around 1990, the fertilizer market at this time is relatively chaotic^49^, resulting in fluctuations in the amount of fertilizer used. The amount of fertilizer application is closely related to the area of arable land. Around 2000, the area of arable land in China decreases, and the amount of fertilization is also affected. However, the use of chemical fertilizers per unit area still causes serious environmental pollution. In 2015, the Ministry of Agriculture formulated and issued the “Action Plan for Zero Growth of Chemical Fertilizer Use by 2020”, which provided new guidance for chemical fertilizer application, which led to a fundamental reduction.

Over the past 40 years, emissions of N and P from manure in the Yangtze River Delta generally increased at first and then decreased. From 1980 to 2005, N and P emissions from manure in the Yangtze River Delta showed an increasing trend. Manure N increased from 2.13 × 10^8^ kg in 1980 to 2.66 × 10^8^ kg in 2005, increased by 24.77%. Manure P increased from 7.34 × 10^7^ kg in 1980 to 1.03 × 10^8^ kg in 2005, increased by 40.11%. After reaching a peak in 2005, emissions showed a continuous declining trend. In 2018, N and P emissions from manure used as organic fertilizer were 1.46 × 10^8^ kg and 5.68 × 10^7^ kg, respectively, back to 1980 levels.

The change trend of manure nitrogen and phosphorus emissions in the Yangtze River Delta is mainly affected by economic development and national policies. From 1985 to 2005, the vigorous economic development also promoted the increasing demand for meat, eggs, and milk in people’s lives. The development of animal husbandry^50^, and there were almost no special regulations on the establishment of sound manure management in the livestock and poultry farming industry before 2000. Factors have contributed to the increase in the discharge of livestock and poultry manure. After 2000, the State Environmental Protection Administration successively promulgated many normative documents, which effectively curb the increase of nitrogen and phosphorus emissions from livestock manure.

#### Spatiotemporal variation characteristics of N and P emissions from livestock manure

**Figure 2:**
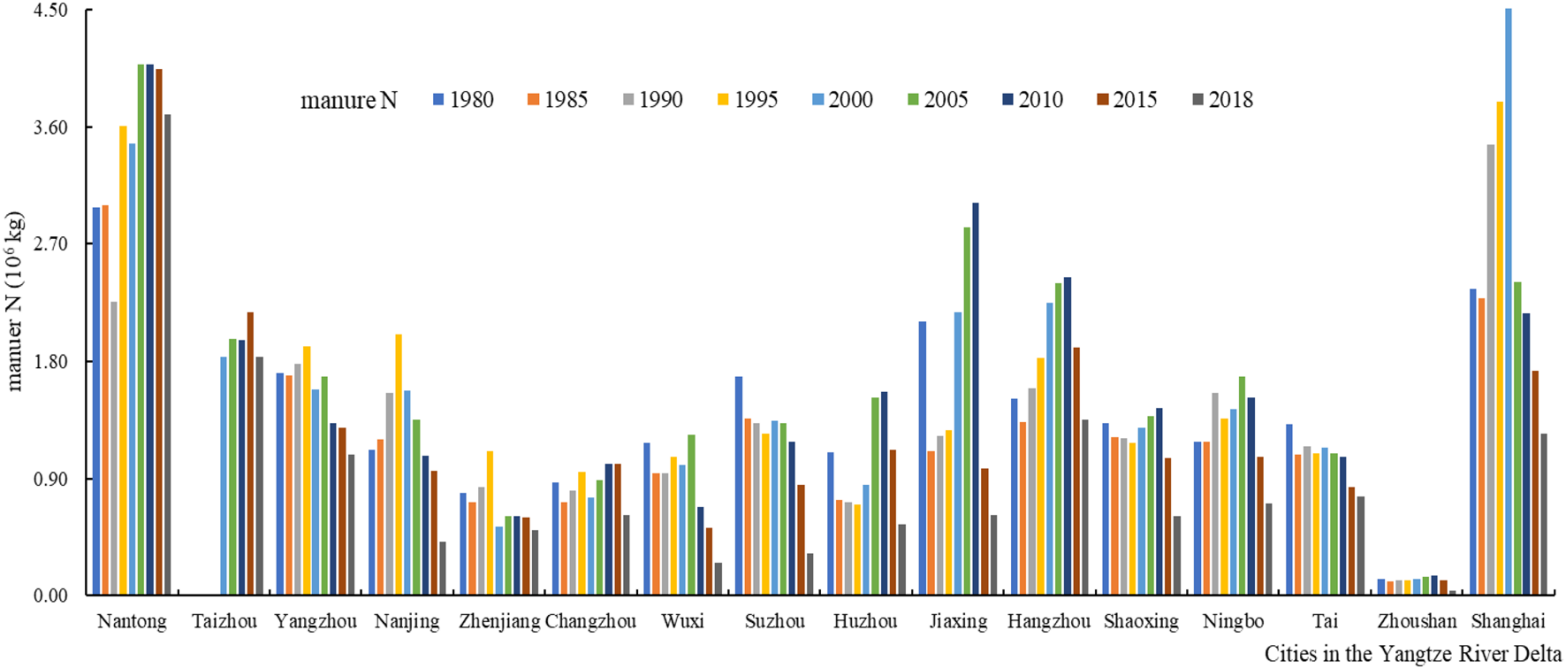
Manure N emissions in cities in the Yangtze River Delta.

**Figure 3:**
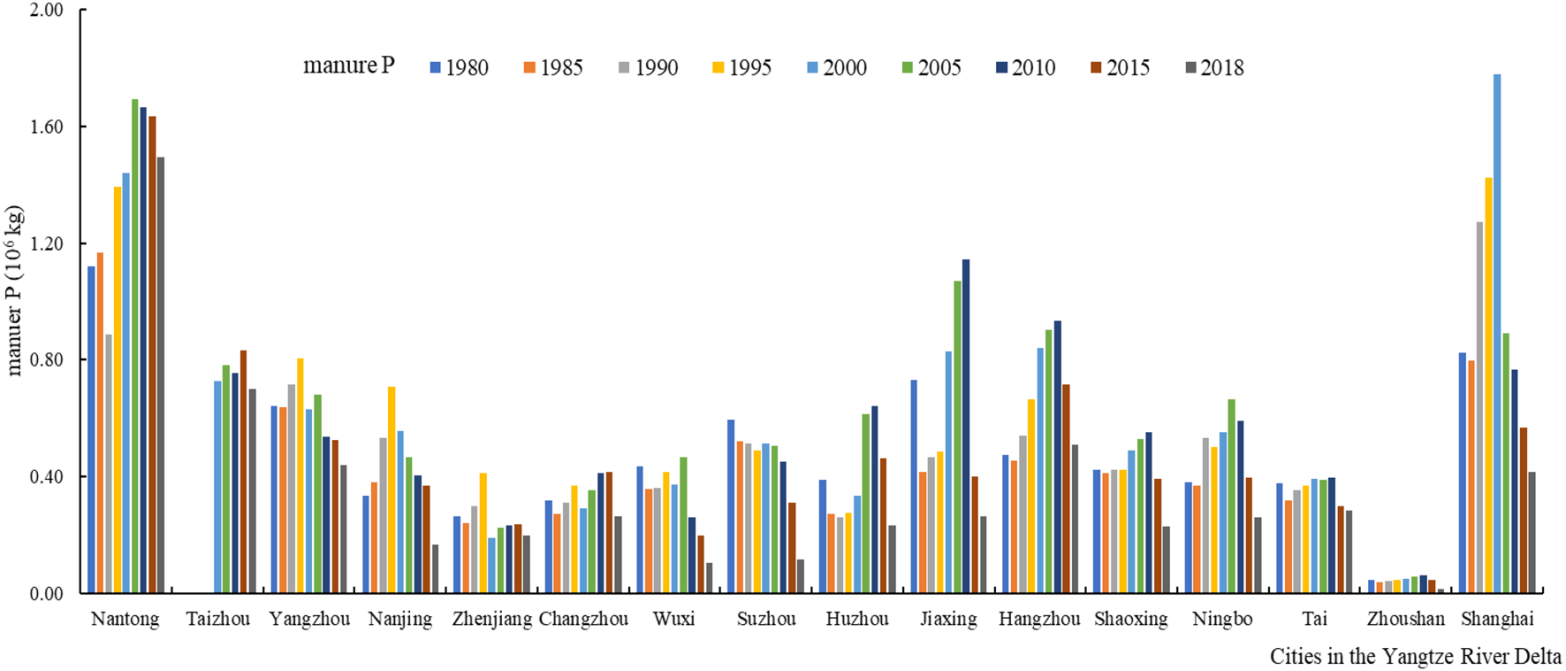
Manure P emissions in cities in the Yangtze River Delta.

There have obvious temporal and spatial variabilities in manure N and P emissions in the soils among cities in the Yangtze River Delta over the past 40 years (Figs. 2, 3). N and P emissions from livestock manure in the soils showed a trend of increasing and then decreasing, with manure nutrient emissions from livestock in some areas showed a fluctuating trend.

Nantong and Shanghai have always been the geographic focus of livestock manure nutrient emissions in terms of spatial change. Average N and P emissions from livestock manure in Nantong over the past 40 years was 3.47 × 10^7^ and 1.39 × 10^7^ kg, respectively; average N and P emissions from livestock manure in Shanghai over the past 40 years was 2.67 × 10^7^ and 9.70 × 10^6^ kg, respectively. During the past 40 years, the total N and P emissions from livestock manure were lowest in Zhoushan. From 1980 to 2018, the average yields of N and P in livestock manure were only 1.18 × 10^6^ and 4.34 × 10^5^ kg, respectively, in Zhoushan. The N and P emissions from livestock manure in other cities fluctuated 3.54 × 10^5^ to 3.01 × 10^7^ kg and 1.35 × 10^5^ to 1.14 × 10^7^ kg, respectively.

Nantong and Shanghai have become the geographic focus of livestock and poultry manure emissions due to the following reasons: on the one hand, rapid economic development, abundant population resources, urban density and high degree of agricultural intensification are all factors that have caused the rapid development of livestock and poultry breeding. On the other hand, arable land resources are extremely scarce, resulting in a unit arable land carrying capacity much higher than other cities, and the dense water network has also accelerated the loss of nitrogen and phosphorus in livestock manure. As Zhoushan is located at the intersection of the golden coastline of eastern China and the golden waterway of the Yangtze River, it is China's largest seafood production base, and its development focus is not on the livestock and poultry breeding industry.

### Changes in manure loads of livestock in the Yangtze River Delta from 1980 to 2018

#### Spatiotemporal pattern of manure N and P loads in livestock

**Figure 4:**
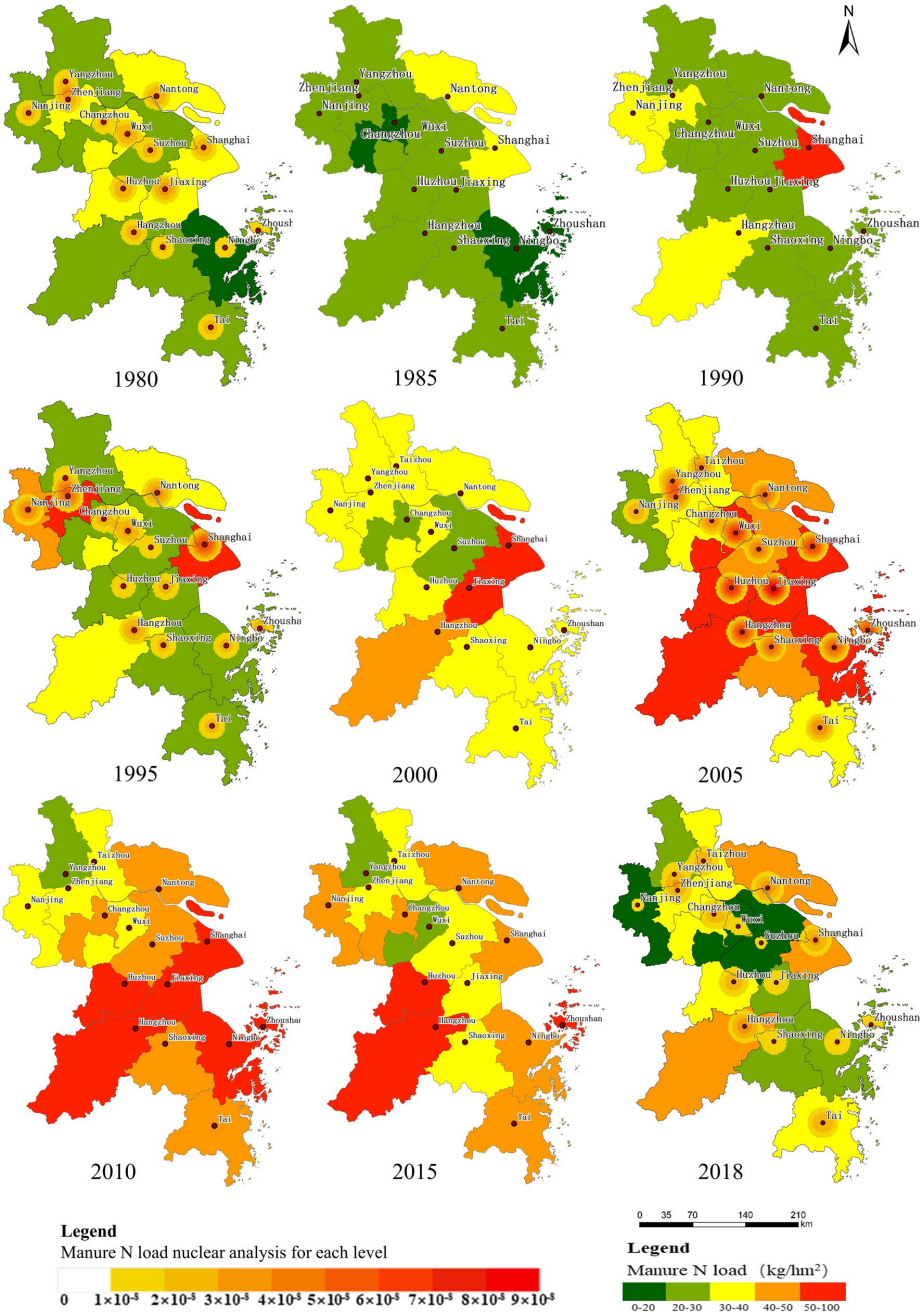
Spatiotemporal distribution of manure N load in the Yangtze River Delta from 1980 to 2018.

From 1980 to 2018, the manure N load in the soils of the Yangtze River Delta showed an overall trend of increasing first and then decreasing (Fig. 4). From 1980 to 2010, most regions of the study area showed an increasing trend in manure N load. In 2010, the on average manure N load was highest. The high load area was rapidly expanding from the north and the middle east to the south of the study area, and the load center shifted from the east to the middle and southwest. From 2010 to 2018, the manure N load decreased year by year, especially in the middle of the study area, and the high-load area transferred to the edge area. In 2018, manure N load recovered roughly to the level seen in the 1990s.

From 1980 to 2010, average manure N load increased from 27.46 to 50.61 kg hm^−2^, representing a growth of 84.30% to the maximum manure N load of the past 40 years. This trend is basically consistent with the gradual increase in the average nitrogen pollution load per unit of arable land in China as shown by the results of earlier studies^48,51–53^. Manure N load in Zhoushan (located in the eastern coastal area), Shanghai, Huzhou, and Jiaxing (located in the central area), and Hangzhou (located in the southwest region) increased significantly: Zhoushan saw the largest increase (295.25%) from 22.04 kg hm^−2^ in 1980 to 87.10 kg hm^−2^ in 2010. On the one hand, the livestock breeding industry is affected by the price regulation and management of the agricultural material market, and on the other hand, it is affected by production price factors. Therefore, the rapid increase in demand for livestock caused by the rapid economic development, the increase in prices, and the increase in the number of breeding industries are the main reasons for the rapid increase in the manure N load during this period. From 2010 to 2018, the average manure loads dropped dramatically, decreasing from 50.61 to 30.29 kg hm^−2^, representing a decline of 40.15%. The average manure N load in Wuxi, Suzhou, Jiaxing (located in the central part), and Zhoushan (located in the eastern coastal area) showed significant declines, with that in Jiaxing decreasing from 100.88 to 24.60 kg hm^−2^, representing a decrease of 75.62%. Feng et al.^54^ found that policy is an important factor that makes livestock breeding industry more standardized and the environment improved. Therefore, the reduction in the manure N load during this period was largely affected by policy regulation. Over the past 40 years, the manure N load in Shanghai has been maintained at a high level, with a load of over 50 kg hm^−2^ throughout the period. By contrast, the manure N load in Yangzhou has been maintained at a low level for a long time, which was below 30 kg hm^−2^, highlighting significant regional differences. The reason is the difference caused by the difference in the degree of urban construction and economic development in different regions. In areas with relatively developed economies, the demand for livestock products is large, and the amount of livestock is large, but the area of arable land is small, and the capacity of absorbing livestock and poultry manure is limited, resulting in a relatively large manure N and P loads on cultivated land^55^.

From 1980 to 2018, the manure N load center moved from the central and northern regions of the study area to the northwestern and eastern, and then to the southwestern and eastern regions after a peak of manure loads was reached in each city. In the 1980s, the maximum livestock manure loads were located in the middle (Jiaxing) and the northern part (Wuxi) of the study area. Subsequently, the center of gravity of manure N emissions from livestock gradually shifted to the east. In the 1990s, the livestock manure N load gravity center was located in the northwestern (Zhenjiang) and eastern (Shanghai) areas. At the beginning of the twenty-first century, the livestock manure N load was maintained at a relatively high level in most cities, and the emission center gradually shifted to the east. In 2018, the livestock manure N load was centred in the southwestern region (Hangzhou) and the eastern region (Nantong, Shanghai).

Considering that there is no systematic standard limit of organic fertilizer N in China, we used the European Union’s farmland manure N limit standard of as the basis for determining the manure N load^51^. From 1980 to 2018, the manure N load did not exceed the European Union’s standard (170 kg hm^−2^), but still showed an increasing trend. It can be seen that the discharge of livestock manure in each city has had adverse impact on the environment of the Yangtze River Delta.

**Figure 5:**
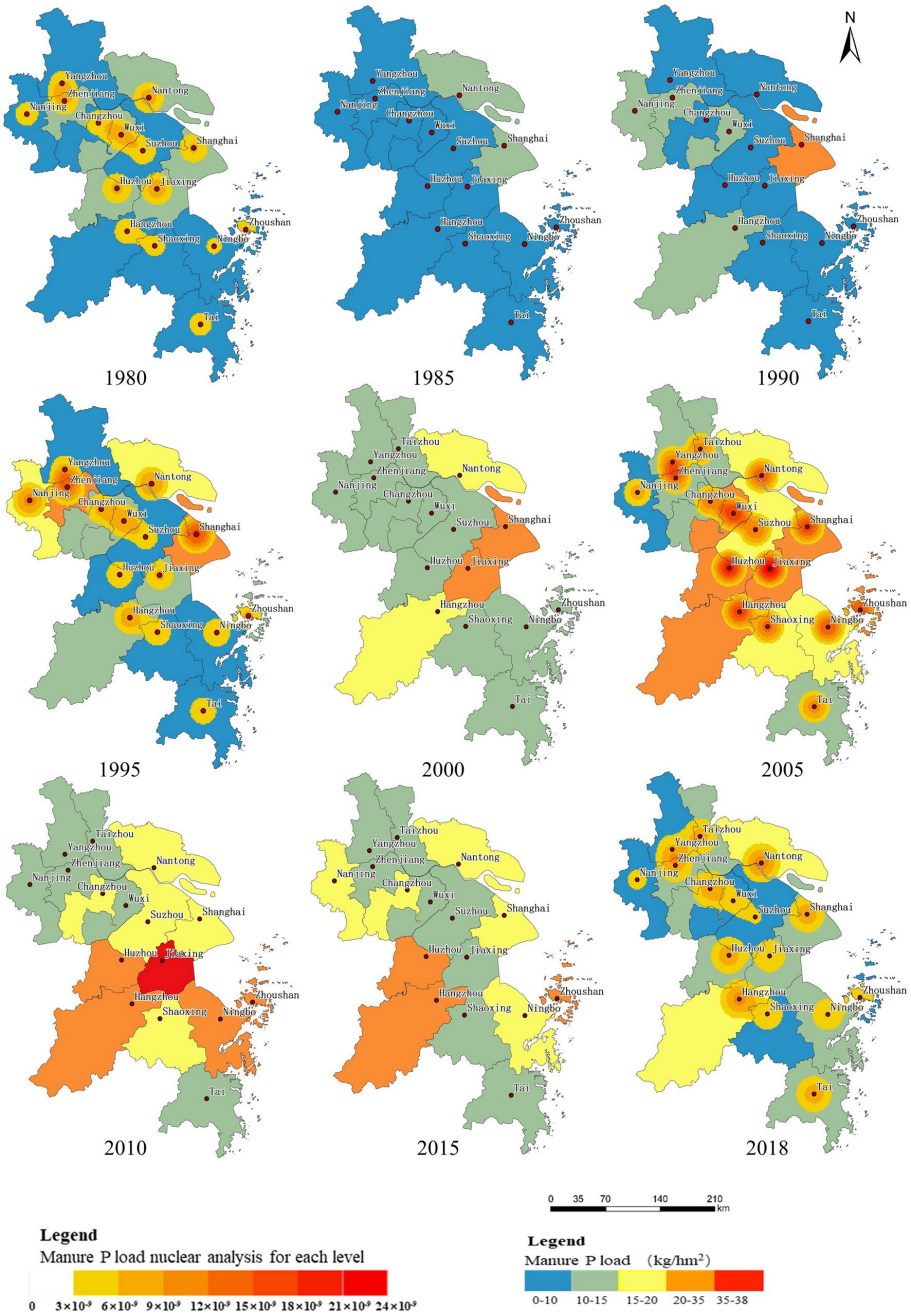
Spatiotemporal distribution of manure P load in the Yangtze River Delta from 1980 to 2018.

From 1980 to 2018, the spatiotemporal evolution pattern of livestock manure P load in the soils was very similar to that of the manure N load, with an overall trend of first increasing and then decreasing (Fig. 5). From 1980 to 2010, the livestock manure P load showed an increasing trend. By 2010, the livestock manure P load had reached its maximum over the past 40 years. Areas with high livestock manure P load spread from the central area to the surrounding cities, and finally radiated to the surrounding regions with the central area as the load center; from 2010 to 2018, the livestock manure P load decreased significantly, and areas with high manure P load migrated from the central region to the southwestern marginal region and the northeastern coastal cities.

From 1980 to 2010, the average livestock manure P load in the Yangtze River Delta increased from 9.36 to 19.47 kg hm^−2^, representing a growth rate of 108.02%. Average manure P loads in Zhoushan, Ningbo (located in the eastern coastal area), and Jiaxing, Hangzhou, and Huzhou (located in the central and southern regions) showed significant increases of 347.66%, 323.28%, 198.13%, 181.88%, and 155.39%, respectively. From 2010 to 2018, the average livestock manure P load was reduced from 19.47 to 11.74 kg hm^−2^, representing a reduction of 39.71%. Zhoushan (located in the eastern coastal area) and Jiaxing and Suzhou (located in the central area) saw the most obvious declines of 73.26%, 72.49%, and 68.05%, respectively. In addition, the average manure P load content was greater than 20 kg hm^−2^ in the central region (Huzhou, Jiaxing, Shanghai) and the southwestern region (Hangzhou), but lower than 15 kg hm^−2^ in the southeastern region (Tai) and northwestern region (Yangzhou, Taizhou).

From 1980 to 2018, the center of the livestock manure P load shifted from the central and northern regions of the study area to the eastern and northwestern regions, and then to the northeastern and southwestern regions after reaching peak P loading in each city. In the 1980s, the livestock manure P load was mainly concentrated in the middle (Jiaxing) and the northern region (Wuxi); in the 1990s, the center of the livestock manure P load moved to the east (Shanghai) and the northwest (Zhenjiang); at the beginning of the twenty-first century, the livestock manure P load remained at high levels in most cities. By 2018, the center of the livestock manure P load had accumulated toward the edge of the Yangtze River Delta, mainly in the northeastern (Nantong) and southwestern regions (Hangzhou).

It is generally believed that annual P application from manure should not exceed 35 kg hm^−2^^56^, otherwise excessive P will result in leaching of soil P and eutrophication of the water body. Livestock manure P load in Jinxing exceeded this standard in 2010, having a certain negative impact on the local environment. The rational treatment and utilization of livestock manure is imminent^57^.

In summary, the Yangtze River Delta’s livestock manure has increased first and then decreased in the past 40 years. The reason for its significant increase before 2010 is mainly due to the urban construction and rapid economic development in various regions, and it spreads to the surrounding areas. With urban development, the area of agricultural land has been greatly reduced, and the area carrying livestock manure nutrient has been relatively reduced, resulting in an increase in the manure N and P load in the Yangtze River Delta. After 2010, the reason for the decrease in the manure N and P load in the Yangtze River Delta may be related to the successive introduction and implementation of environmental protection policies for livestock breeding in various provinces and cities. After the promulgation of the “Pollution Prevention and Control Technology Policy for Livestock and Poultry Breeding Industry” in 2010, the state has strengthened the macro-control of the livestock breeding industry, the Yangtze River Delta has been listed as a restricted development zone, the industrial structure has been further optimized and adjusted, and the amount of livestock breeding has been significantly reduced. This leads to a decrease in the manure N and P load in the Yangtze River Delta. The manure N and P load has been weakened year by year. This change pattern is also consistent with the trend of policy measures. To a certain extent, it shows that the current livestock and poultry pollution prevention and control measures have achieved remarkable results^58^.

#### Changes in livestock manure N and P loads from 1980 to 2018

**Figure 6:**
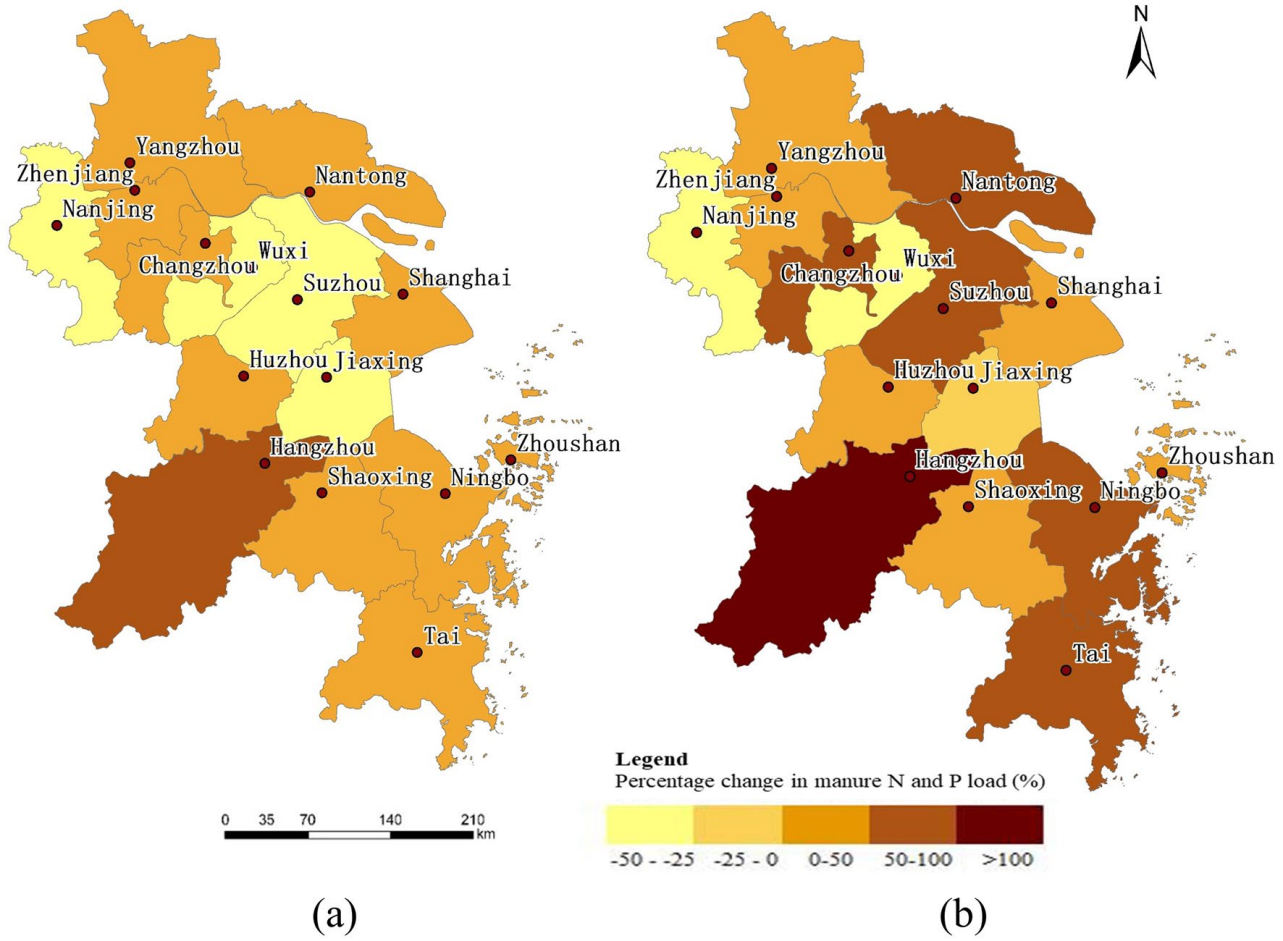
(**a**) Percentage change of manure N loads from 1980 to 2018; (**b**) Percentage change of manure P loads from 1980 to 2018.

Between 1980 and 2018, manure N and P loads showed significant spatial variability (Fig. 6). Due to the influence of government guidance, large demand for production land, and environmental protection pressure, changes to animal husbandry space have been promoted^59–61^. Livestock manure N and P loads in the northwestern and central regions have decreased significantly, while manure N and P loads in the surrounding areas have increased to varying degrees, showing a general shift from the central region to the surrounding cities. Specifically, in 2018, the manure N and P loads in Nanjing, Wuxi, Suzhou, and Jiaxing showed a decreasing trend. Compared with 1980, the manure N load decreased by 41.55%, 48.26%, 44.49%, and 33.46%, respectively. Compared with 1980, the manure P load decreased by 21.63%, 43.08%, 43.47%, and 17.98%, respectively. The reduction in manure N and P loads in the central region is related to policies of livestock pollution prevention, which were successively promulgated in the Tai Lake area^32,33,62^. The relevant departments have optimized the regional layout of animal husbandry and comprehensively made use of the livestock manure to reduce pollution, thereby reducing the manure N and P loads. All the 11 cities of Yangzhou, Zhenjiang, Changzhou, Nantong, Shanghai, Huzhou, Hangzhou, Shaoxing, Ningbo, Zhoushan, and Tai saw different degrees of increase in their manure N and P loads, and Hangzhou increased most. Compared with 1980, the manure N and P load of Hangzhou in 2018 increased by 76.72% and 112.58%, respectively. The manure N and P loads in the remaining 10 cities increase less than 100%. Increases in manure N and P loads may be related to the small scale^63,64^ and scattered distribution of local farms, and lack of environmental protection awareness among residents^65,66^.

### Identification of high-risk areas of soil pollution caused by livestock manure

**Figure 7:**
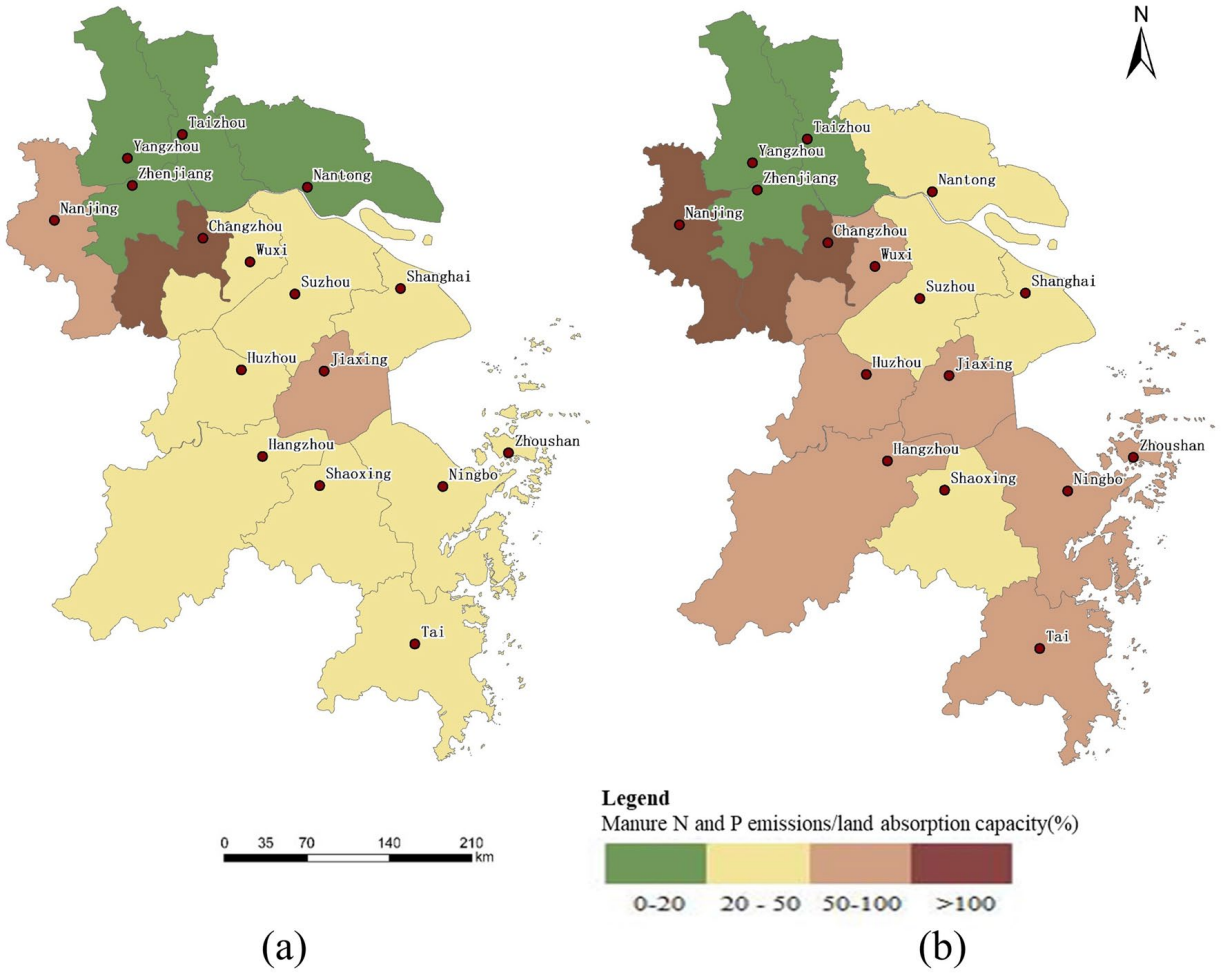
(**a**) Manure N emission relative to land absorption capacity in 2018; (**b**) manure P emission relative to land absorption capacity in 2018.

The high-risk areas for manure N and P emissions in 2018 were mainly located in the northwestern and southern regions of the Yangtze River Delta (Fig. 7), while the manure N and P emissions in some northern cities could not meet the nutrient requirements of the local land. Manure N and P emissions in Changzhou were 215.60% and 334.54% of the land’s absorption capacity, while those in Nanjing were 102.18% and 71.02%, respectively. It shows that the imbalance between the supply and demand of planting and breeding may cause a greater risk of environmental pollution of livestock and poultry, and it is necessary to reduce the scale of breeding or expand the scale of planting^67,68^. Manure P emissions in Wuxi, Huzhou, Jiaxing, Hangzhou, and Zhoushan were close to the maximum land absorption capacity for livestock manure nutrients, indicating that the supply and demand for planting and breeding were balanced. Therefore, the use of local organic fertilizers can be appropriately increased to reduce the amount of chemical fertilizers used and reduce the potential pollution threat caused by the enrichment of manure nutrients^69,70^. In the northern region, the discharge of livestock manure in Yangzhou, Taizhou, Nantong, and Zhenjiang was only 0–20% of the land absorption capacity, indicating that livestock manure nutrient in these areas cannot meet the nutrient needs of local crops. Therefore, additional nutrient supply is needed to meet the normal growth of local crops^71^.

### Selection of typical models and main control factors based on long-term manure N and P emissions

#### Systematic clustering analysis of manure N and P emissions

**Figure 8:**
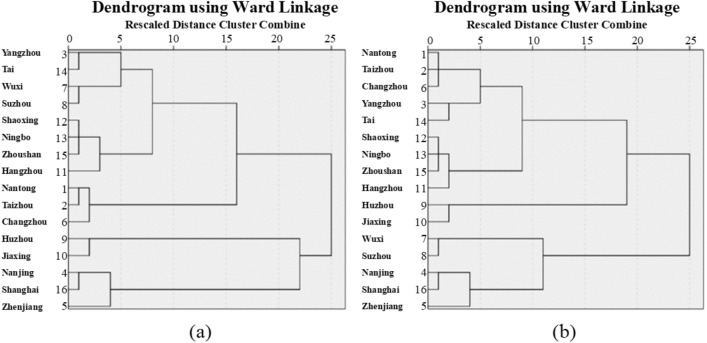
(**a**) Systematic clustering of manure N emissions from 1980 to 2018; (**b**) systematic clustering of manure P emissions from 1980 to 2018.

According to the similarity of manure N and P emissions in the cities, we carried out variable analysis based on the Ward minimum variance method^72^. Cities were divided into four categories based on the change trend of manure N emissions in the Yangtze River Delta^73^ (Fig. 8a). Class I: Yangzhou, Tai, Wuxi, Suzhou, Shaoxing, Ningbo, Zhoushan, Hangzhou; class II: Nantong, Taizhou, Changzhou; class III: Huzhou, Jiaxing; class IV: Nanjing, Shanghai, Zhenjiang.

Similar to the manure N classification method, cities were divided into four categories based on the change trend of manure P emissions in the Yangtze River Delta (Fig. 8b). Class I: Nantong, Taizhou, Changzhou, Yangzhou, Tai, Shaoxing, Ningbo, Zhoushan, Hangzhou; class II: Huzhou, Jiaxing; class III: Wuxi, Suzhou; class IV: Nanjing, Shanghai, Zhenjiang.

#### Principal component analysis of manure N and P emissions

Based on the results of systematic clustering, typical cities were extracted to establish a typical model of manure N and P emissions and the main control factors were selected^74^. For manure N emissions, Yangzhou, Nantong, Huzhou, and Shanghai were selected from Class I, Class II, Class III, and Class IV, respectively. Combining these with the rising and falling trend characteristics of manure N emissions over the long study period, we established four typical models of manure N emissions as “up-down-down” model, “down-up-up” model, “down-up-down” model, and “up-up-down” model. According to the clustering results of manure P emissions, Hangzhou, Jiaxing, Suzhou, and Shanghai were selected from Class I, Class II, Class III, and Class IV, respectively. Four typical models of manure P emissions were established based on rising and falling trend characteristics of manure N emissions over the long study period as “up-up-down” model, “down-up-down” model, “down-level-down” model, and “up-down-down” model.

#### Analysis on the main control factors for manure N emissions

**Figure 9:**
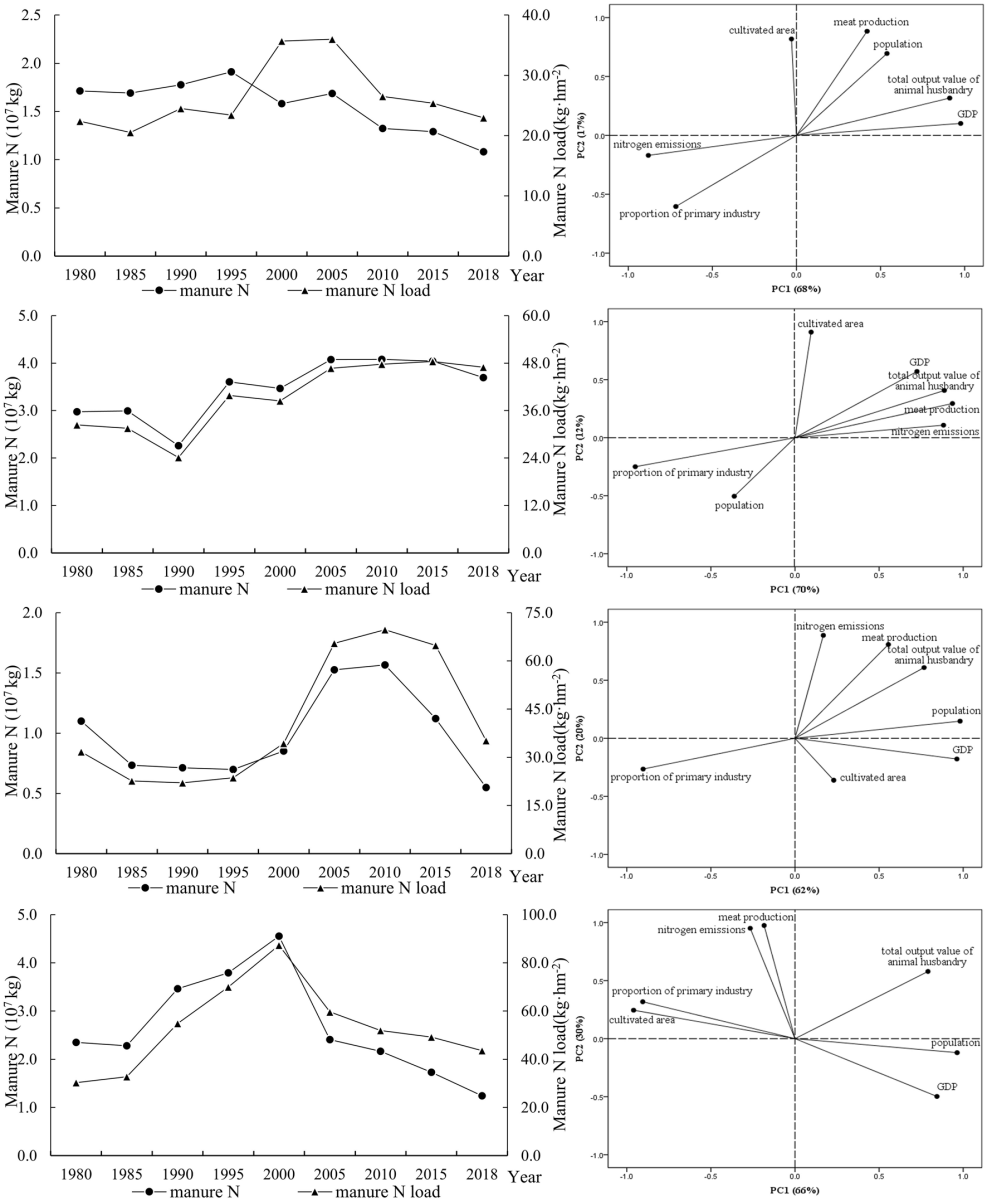
Manure N emissions from 1980 to 2018 and main control factors based on principal component analysis.

For the “up-down-down” model in Yangzhou, the total variance of the two principal components accounted for 85% (Fig. 9a); Nantong was characterized as a typical “down-up-up” model city, where the variance of the two principal components ac-counted for 82% (Fig. 9b); Huzhou represented a typical “down-up-down” model city, where the sum of the variances of the two principal components was 82% (Fig. 9c); in the “up-up-down” model for Shanghai, the sum of variance of the two principal com-ponents was 96% (Fig. 9d).

There was a significant positive correlation between changes in manure N emissions and the proportion of the primary industry in Yangzhou, indicating that the “up-down-down” model of Class I is mainly affected by the primary industry. The “Pollution Prevention and Control Plan for Livestock Breeding Industry in Yangzhou” proposes to delimit forbidden and restricted areas, regulate livestock breeding, and reduce pollutant discharge from livestock breeding. Therefore, the scale of livestock breeding in Yangzhou has decreased, and the total manure N has shown a downward trend, which is consistent with the interannual changes in the proportion of the primary industry. The primary industry in this class is mainly agriculture and animal husbandry; thus, the main control factor for the total manure N is the proportion of primary industry.

The total manure N in Nantong first decreased and then increased, and finally tended to be flat over the study period. There was a clear correlation between changes in manure N emissions in Nantong, meat production, and the total output value of animal husbandry, indicating that Class II is dominated by these two factors. The livestock breeding industry in Class II is relatively developed^75,76^, and meat production showed a consistent trend with total manure N. Hence, meat production and the total output value of animal husbandry are the factors having the greatest impact on Class II.

Total manure N in Huzhou showed a trend of decreasing, then increasing, and finally decreasing. Manure N emissions changes and meat production showed a relatively obvious positive correlation, indicating that Class III is mainly affected by meat production and has little correlation with factors such as GDP, which is consistent with the changing trend of meat production. Huzhou’s agriculture is dominated by planting and fishery^77^, and the impact of animal husbandry is not significant, so meat production is the factor having the greatest impact on such cities.

Total manure N in Shanghai began to increase over the study period, consistent with changes in meat production. There was a strong positive correlation between changes in manure N emissions and meat production, indicating that Class IV is greatly affected by arable land area. After that, Shanghai issued relevant measures to regulate livestock breeding, such as the “Shanghai Livestock a Breeding Management Measures”. Due to a decline in farmland area, meat production decreased, and manure N showed a downward trend^78^. Total manure N and meat production in Shanghai both increased at first and then decreased. Therefore, meat production is the most influential factor in such cities.

#### Selection of main control factors for manure P emissions

**Figure 10:**
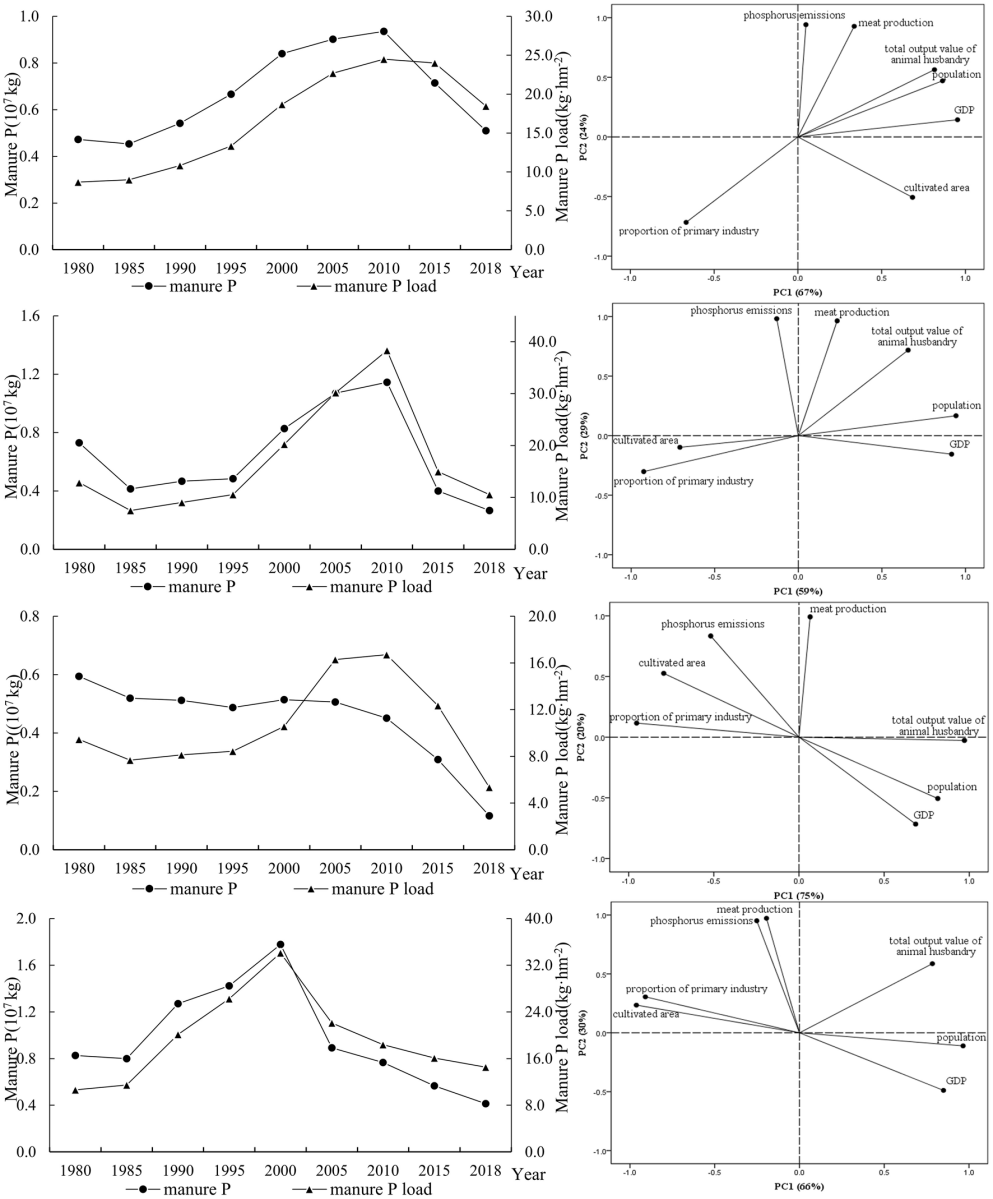
Manure P emissions from 1980 to 2018 and main control factors based on principal component analysis.

In the “up-up-down” model for Hangzhou, the total variance of the two principal components accounted for 91% (Fig. 10a); as a typical “down-up-down” model, the total variance of the two principal components in Jiaxing accounted for 88% (Fig. 10b); Su-zhou represented a typical “down-flat-down” model, and the sum of the variance of the two principal components accounted for 95% (Fig. 10c); as an example of the “up-down-down” model, the variance of the two principal components in Shanghai accounted for 96% (Fig. 10d).

Manure P in Hangzhou first increased and then decreased, essentially the same as the trend for Hangzhou’s meat production. There was a strong positive correlation between changes in manure P emissions and the total meat production in Yangzhou, indicating that the “up-up-down” model of Class I is mainly affected by meat production. Due to the serious pollution from livestock and poultry in Hangzhou^79^, relevant policies have been introduced to reduce the amount of livestock breeding, thereby reducing meat production.

Manure P in Jiaxing showed a trend of first decline, then rise, and finally decline. There was a large positive correlation between changes in manure P emissions and meat production in Jiaxing, indicating that the “down-up-down” model of Class II is mainly affected by meat production. Class II is dominated by agriculture and animal husbandry^34,35^, and the breeding industry is relatively developed. Meat production also changes with these industries, and its inter-annual variation is consistent with that of manure P.

Manure P in Suzhou showed a downward trend, consistent with inter-annual changes in the area of arable land. Changes in manure P emissions and the area of arable land showed a significant positive correlation, indicating that the Class III cities with a “decrease-level-decrease” model were mainly affected by the area of arable land and the proportion of the primary industry. Suzhou is an industrial development base that cannot be ignored. Local economic development is relatively rapid^80^, so its arable land area is continuously decreasing^81^.

The livestock manure P in Shanghai increased at first and then decreased, which is consistent with inter-annual changes in meat production. In 2002, the Shanghai Municipal People’s Government highlighted a special plan for Shanghai’s animal husbandry, stipulating prohibition of breeding areas, control of breeding areas and moderate breeding areas. The city’s total livestock and poultry production decreased, and meat production decreased^82^. There was a strong positive correlation between the changes in livestock manure P emissions and meat production, indicating that the “up-down-down” model of Class IV is mainly affected by meat production.

## Conclusions

This study initially explored the characteristics of temporal and spatial changes in manure N (P) and manure N (P) loads in the soils from 1980 to 2018 in the Yangtze River Delta and identified high-risk areas for livestock manure pollution. From 1980 to 2018, the livestock manure N (P) and manure N (P) load in Yangtze River Delta cities showed a trend of first increasing and then decreasing over time. The manure N (P) load will eventually shift from the central part of the Yangtze River Delta to the northeast and southwest. Given the ability of land in the Yangtze River Delta to absorb nutrient from livestock manure in 2018, we identified Changzhou as a high-risk area for livestock manure pollution. For the Yangtze River Delta, on the one hand, organic fertilizers can be used instead of chemical fertilizers to reduce the potential threat of manure nutrient enrichment. On the other hand, effective manure nutrient management can be carried out by adjusting the livestock population structure.

Through clustering and PCA, we established typical models of manure N and P emissions and the main factors control them. The typical models of manure N emissions from livestock are “up-down-down”, “down-up-up”, “down-up-down”, and “up-up-down”. The main control factors are the proportion of meat production and primary industry. Typical patterns of manure P emissions are “up-up-down”, “down-up-down”, “down-flat-down”, and “up-down-down”. The main controlling factors are meat class yield and cultivated land area.

In future research, we will further combine micro-mechanism studies to explore the effects of manure N and P emissions on the N and P cycles of water bodies, and provide references for exploring a balanced model of planting and feeding and evaluating pressure on the ecological environment.

## Data Availability

Data is available upon request by contacting the corresponding author.
